# Pure Laparoscopic Living Donor Hepatectomy With/Without Fluorescence-Assisted Technology and Conventional Open Procedure: A Retrospective Study in Mainland China

**DOI:** 10.3389/fsurg.2021.771250

**Published:** 2021-12-13

**Authors:** Kang He, Yixiao Pan, Hai Wang, Jianjun Zhu, Bijun Qiu, Yi Luo, Qiang Xia

**Affiliations:** Department of Liver Surgery, Renji Hospital Affiliated to Shanghai Jiao Tong University School of Medicine, Shanghai, China

**Keywords:** pure laparoscopic living donor hepatectomy, fluorescence-assisted technology, open donor hepatectomy, quality of life, living donor liver transplantation

## Abstract

**Background:** The application of laparoscopy in donor liver acquisition for living donor liver transplantation (LDLT) has become increasingly popular in the past decade. Indole cyanide green (ICG) fluorescence technique is a new adjuvant method in surgery. The purpose was to compare the safety and efficacy of laparoscopic and open surgery in living donor left lateral hepatectomy, and to evaluate the application of ICG in laparoscopy.

**Methods:** Donors received LDLT for left lateral lobe resection from November 2016 to November 2020 were selected and divided into pure laparoscopy donor hepatectomy (PLDH) group, fluorescence-assisted pure laparoscopy donor hepatectomy (FAPLDH) group and open donor hepatectomy (ODH) group. We compared perioperative data and prognosis of donors and recipients. Quality of life were evaluated by SF-36 questionnaires.

**Results:** The operation time of PLDH group (169.29 ± 26.68 min) was longer than FAPLDH group (154.34 ± 18.40 min) and ODH group (146.08 ± 25.39 min, *p* = 0.001). The blood loss was minimum in FAPLDH group (39.48 ± 10.46 mL), compared with PLDH group (52.44 ± 18.44 mL) and ODH group (108.80 ± 36.82 mL, *p*=0.001). The post-operative hospital stay was longer in PLDH group (5.30 ± 0.98 days) than FAPLDH group (4.81 ± 1.03 days) and ODH group (4.64 ± 1.20 days; *p* = 0.001). Quality of life of donors undergoing laparoscopic surgery was better.

**Conclusion:** Laparoscopic approaches for LDLT contribute to less blood loss, better cosmetic satisfaction. The fluorescence technique can further reduce bleeding and shorten operation time. In terms of quality of life, laparoscopic surgery is better than open surgery. Laparoscopy procedure for living-donor procurement with/without fluorescence-assist can be performed as safely as open surgery.

## Introduction

In the past 100 years, with the rapid development of surgery and the emergence of new immune agents, liver transplantation has become an important or even the only treatment method for diverse end-stage liver diseases (1). Due to the shortage of deceased donor organs, living donor liver transplantation (LDLT) has become the most important surgical method to replace cadaveric donor livers, and LDLT has been proven to have at least the same prognosis as cadaveric donor liver transplantation (2). It is worth noting that since most of the living donor liver donors are healthy adults, the safety of the donors has also become a focus of attention. However, the huge surgical risk of hepatectomy is a major problem faced by surgery operators. It has been reported that the incidence of donor complications can reach 20–40% (3–7), including even some deaths of donors (8). Although open donor hepatectomy (ODH) is considered a safe surgery operation, a large study shows that 38% of ODH donors have complications of varying degrees, such as incisional hernia, incision numbness, long-term abdominal discomfort, and mental illness (9–11). This suggests that we need to find a safer and more effective surgical method.

For a long time, open donor hepatectomy has been considered a traditional liver transplantation operation. However, in the past two decades, the proportion of laparoscopic applications in liver surgery has continued to grow. In the 2014 Morioka Declaration, it was mentioned that although laparoscopic surgery is not a mainstream liver surgery method, small-range hepatectomy under laparoscopy has become a routine surgical option (12). In addition, the efficacy and safety of laparoscopic hepatectomy for liver tumors have also been well-confirmed (13, 14). Since Cherqui et al. first reported pure laparoscopic living donor liver transplantation in 2002 (15), the application of pure laparoscopic living donor hepatectomy (PLDH) has been carried out in many transplant centers around the world. Unfortunately, the use of PLDH is still controversial. Although some research groups have reported the effectiveness and safety of this procedure (16–19), there is still a lack of comprehensive, multi-center, large sample, and prospective studies of PLDH. In addition, PLDH requires the operator to have rich surgical experience and skilled laparoscopic operation techniques, which makes PLDH difficult to popularize and so that its development is relatively slow.

In the context of the prevalence of the concept of minimally invasive surgery, laparoscopy is considered to be an effective means to reduce complications after LDLT such as abdominal wall injury, abdominal wall hernia, and intestinal obstruction (20). From the current research reports, compared with traditional ODH, PLDH has the advantages of less intraoperative bleeding, less post-operative pain, shorter recovery time, and lower surgical complication rate (16–19). And the cosmetic advantage brought by smaller wounds is also favored by female donors (16).

In recent years, indocyanine green (ICG), as a near-infrared fluorescent dye, since it was first used by Ishizawa et al. to guide hepatectomy (21), the use of ICG molecular fluorescence imaging technology in liver tumor diagnosis and surgical navigation has been gradually increasing. ICG can be excited by light with a wave length range of 750–810 nm and emit near-infrared light with a wavelength of about 840 nm (22). After intravenous injection of ICG, it quickly binds to plasma proteins, which is almost completely absorbed by hepatocytes, and then excreted through bile (23). Compared with methyl blue or indigo carmine, which usually just stays in the blood vessels of the liver, ICG fluorescent technology is more like a functional staining of the liver. And unlike the short stay time of traditional staining, ICG fluorescent staining can be retained in the liver for several hours. In LDLT, ICG has two main applications: (1) Bile duct imaging, which can clearly obtain bile duct anatomy, and it is helpful for determining the donor liver pre-resection line and bile duct cutting point during the operation; (2) Assessing the patency of the blood vessel after reconstruction and the recovery of liver function of the transplanted liver. Therefore, we believe that ICG technology combined with PLDH is a very promising surgical method, which may have great significance for the safety and prognosis of the donor.

This article retrospectively analyzed the effects of OLDH, PLDH, and FAPLDH approaches in LDLT operation on donors and their recipients, and explored the effectiveness and safety of PLDH and ICG technology (FAPLDH) in LDLT.

## Methods

### Patients Population and Study Design

The study was conducted in accordance with the Declaration of Helsinki (as revised in 2013). This study has been approved by the Institutional Review Board of Renji Hospital affiliated to Shanghai Jiao Tong University School of Medicine (IRB Reference Number: KY2020-190).

From November 2016 to November 2020, all donors who have received left lateral lobe living hepatectomy for liver transplantation in Shanghai Renji Hospital by laparoscopy or fluorescence-assisted laparoscopy were included. In addition, we randomly selected a portion of open left lateral lobe living hepatectomy for liver transplantation in the same period as the control group. Exclusion criteria were patients lost to follow-up. All donors and recipients were divided into three groups: PLDH group (donors 90 cases, recipients 90 cases), FAPLDH group (donors 58 cases, recipients 58 cases), and traditional ODH group (donors 137 cases, recipients 137 cases). All operations were performed by surgeons experienced in living donor hepatectomy and laparoscopic hepatectomy. In this study, all operations were performed continuously by the same surgical team. All demographic and medical data were obtained from medical records. In addition, SF-36 questionnaire follow-up was conducted among the donors who underwent open surgery (218 cases) and laparoscopic surgery (119 cases) at the same period, to further compare the effects of open surgery and laparoscopic surgery on the prognosis and quality of life of the donors.

### Donor Assessment and Surgical Techniques

Pre-transplant evaluation of donors included blood routine test, liver and kidney function tests, testing for hepatitis, EBV and CMV, Doppler ultrasonography, triphasic liver computed tomography (CT) with volumetry, and magnetic resonance cholangiopancreatography (MRCP). Percutaneous biopsy of liver is needed if severe steatosis is suspended. Donors and their families were given a thorough explanation of the advantages and disadvantages of open and laparoscopic donor hepatectomy, after which they decided on their preferred type of surgery. For open donor surgery, abdomen was explored using a middle line skin incision and the left lateral sectionectomy with donor gallbladder preservation was performed as described. For laparoscopic surgery, donor was placed supine in the 30° reversed Trendelenburg position, with the camera holder standing between the donor's legs. No Pringle maneuver was used during parenchymal division. The left bile duct was exposed after partial division of the remnant hepatic parenchyma, and was cut just above a Hem-o-lok clip that was clipped on to the proposed target level of the left hepatic duct in terms of MRCP result. However, for FAPLDH group, 15 min before cutting the left bile duct, 1 ml ICG (2.5 mg/ml) was bolus injected intravenously. The biliary confluence and left hepatic duct were then clearly visualized and sectioned. After infusion of 5,000 units of heparin, the proximal end of the left hepatic artery and left portal vein was clipped and divided. A linear stapler was used to cut the left hepatic vein. The graft was placed in an endobag inserted through a 12-mm trocar and retrieved through a 10-cm suprapubic incision site. The graft was flushed on the back table with 1 liter of UW solution at 4°C through the left portal vein for implantation. Both PLDH and FAPLDH group were using the an ICG fluorescence probe-mounted laparoscopic system (Pinpoint, Stryker, USA) which contains can acquire and display high-definition white light and near-infrared fluorescence images in real-time.

### Questionnaires

The MOS item short from health survey SF-36, was based on the medical outcome study scale (medical outcomes study –short from, MOS SF) invented by Stewartse in 1988, developed by the Boston Health Study in the United States. 1991 The Chinese version of SF-36 was translated by the Department of Social Medicine of Zhejiang University School of Medicine.

As a concise health questionnaire, SF-36 comprehensively summarized the quality of life of the respondents from eight aspects including physical functioning, role-physical, bodily pain, general health, vitality, social functioning, role-emotional and mental health. In addition to the above 8 aspects, the SF-36 also contains another measure of Health: Reported Health Transition (HT), which is used to evaluate overall changes in Health over the past year.

1. PF: Physical Functioning- Measure whether health condition interferes with normal physical activity.
2. RP: Role-Physical- Measure role limitations due to physical health problems.
3. BP: Bodily Pain- Measure the degree of pain and its effect on daily activities.
4. GH: General Health- Measure the individual's evaluation of their own health status and its development trend.
5. VT: Vitality- Measure individuals' subjective perception of their vitality and fatigue levels.
6. SF: Social Functioning- Measure the impact of physical and psychological problems on the quantity and quality of social activities and evaluate the effect of health on social activities.
7. RE: Role-Emotional- Measure role limitations due to emotional problems.
8. MH: Mental Health- Measure four types of mental health item, including motivation, depression, behavioral or emotional loss of control, and psychological subjective feelings.
9. HT: Reported Health Transition- Evaluate overall changes in health status during the past year.

### Statistical Analysis

Continuous data were presented as mean ± standard deviation or median (interquartile range, IQR), and compared by analysis of variance (ANOVA) and Bonferroni's multiple comparisons (for donor and recipient statistics), independent sample Student *t*-test (for the outcome of SF-36). Categorical data were presented as ratio and compared by χ^2^ test. All statistical analyses were performed using SPSS version 23.0 statistical software (IBM SPSS, Chicago, IL, USA). *P*-values < 0.05 were considered statistically significant.

## Results

Of the 285 living transplant donors (Table 1), 90 (32%) underwent pure laparoscopic surgery, 58 (20%) underwent fluorescence-assisted laparoscopic surgery, and 137 (48%) underwent conventional open surgery. There were no aborted operations, no conversion from laparoscopy to open surgery. In these three groups, the basic characteristics [age, sex, height, body weight, body mass index (BMI)], pre-operative data (pre-operative AST, ALT, TB, PT and INR), intraoperative data (graft weight, GRWR, intraoperative blood loss, operating time), post-operative results (length of hospital stay, complications, post-operative peak AST, ALT, TB, PT and INR), and the prognosis of corresponding recipients (albumin of the first day after operation, post-operative peak AST, ALT, TB, PT and INR, post-operative complications, ICU time) were counted and analyzed (Table 2).

**Table 1:** Donors' baseline characteristics and outcomes.

**Analyzed factors**	**ODH (*n* = 137)**	**PLDH (*n* = 90)**	**FAPLDH (*n* = 58)**	***p*-value**
Sex (male:female)	61:76	20:70	12:46	0.001^*&^
Age (years)	30.29 ± 4.49	29.07 ± 4.64	29.88 ± 4.33	0.135
Height (cm)	165.42 ± 8.48	163.16 ± 6.44	164.00 ± 6.32	0.075
Weight (kg)	61.54 ± 11.71	57.12 ± 10.21	58.46 ± 10.42	0.01*
BMI (kg/m^2^)	22.33 ± 2.81	21.39 ± 3.13	21.62 ± 2.70	0.04*
Pre-operative ALT (IU/L)	22.84 ± 9.91	20.90 ± 7.65	23.17 ± 7.57	0.188
Pre-operative AST (IU/L)	19.00 ± 7.10	18.82 ± 6.75	20.12 ± 6.06	0.483
Pre-operative TB (umol/L)	11.68 ± 4.60	12.29 ± 4.48	12.80 ± 4.03	0.248
Pre-operative PT (s)	10.79 ± 0.74	10.68 ± 0.81	10.84 ± 0.78	0.434
Pre-operative INR	0.97 ± 0.07	0.96 ± 0.07	0.98 ± 0.07	0.475
Operation time (min)	146.08 ± 25.39	169.29 ± 26.68	154.34 ± 18.40	0.001^*#^
Intraoperatve blood loss (ml)	108.80 ± 36.82	52.44 ± 18.44	39.48 ± 10.46	0.001^*#&^
Graft weight (g)	270.25 ± 71.33	239.46 ± 41.07	241.38 ± 42.22	0.001^*&^
Post-operative hospitalization (days)	4.64 ± 1.2	5.30 ± 0.98	4.81 ± 1.03	0.001^*#^
Post-operative peak ALT(IQR) (IU/L)	181.00 (111.05–291.00)	160.50 (101.00–336.25)	123.50 (80–228)	0.104
Post-operative peak AST(IQR)(IU/L)	132.00 (81.50–226.00)	155.50 (91–282)	119.00 (77.00–201.25)	0.212
Post-operative peak TB (umol/L)	21.96 ± 10.08	21.87 ± 10.96	18.64 ± 7.58	0.082
Post-operative peak PT(s)	12.81 ± 1.09	12.88 ± 1.54	12.42 ± 0.88	0.061
Post-operative peak INR	1.14 ± 0.11	1.16 ± 0.14	1.12 ± 0.08	0.099

*Significant difference between ODH and PLDH; ^#^significant difference between PLDH and FAPLDH; ^&^significant difference between ODH and FAPLDH. Values are mean (±s.d.) unless indicated otherwise. BMI, body mass index; ALT, alanine aminotransferase; AST, aspartate aminotransferase; TB, total bilirubin; PT, prothrombin time; INR, international normalized ratio; IQR, interquartile range.

**Table 2:** Recipients' baseline characteristics and outcomes.

**Analyzed factors**	**ODH (*n* = 137)**	**PLDH (*n* = 90)**	**FAPLDH (*n* = 58)**	***p*-value**
Age (months)	9.95 ± 8.63	8.87 ± 5.08	8.22 ± 5.92	0.253
Height (cm)	68.87 ± 9.19	67.49 ± 5.24	67.79 ± 6.63	0.37
Weight (kg)	7.85 ± 2.30	7.57 ± 1.42	7.58 ± 1.55	0.47
BMI (kg/cm^2^)	16.39 ± 2.00	16.54 ± 1.81	16.45 ± 2.03	0.846
Sex (male/female)	65:72	51:39	22:36	0.08
GRWR (%)	2.99 ± 0.93	3.26 ± 0.78	3.19 ± 0.80	0.056
ALB (g/L)	30.56 ± 5.16	29.54 ± 5.45	29.70 ± 5.07	0.298
ALT (IU/L)	595.51 ± 732.74	715.29 ± 644.64	564.5 ± 546.28	0.308
AST (IU/L)	568.89 ± 487.88	671.89 ± 414.91	604.72 ± 495.72	0.269
TB (umol/L)	114.35 ± 92.12	130.96 ± 67.36	106.89 ± 72.79	0.163
PT (s)	24.44 ± 8.07	25.48 ± 5.34	24.14 ± 4.99	0.407
INR	2.20 ± 0.77	2.28 ± 0.49	2.18 ± 0.46	0.530
Post-operative hospitalization (days)	19.12 ± 8.70	18.80 ± 7.14	17.95 ± 4.28	0.611
Post-operative ICU time (days)	4.97 ± 1.35	4.84 ± 0.97	5.05 ± 1.09	0.556
Post-operative death (*n*)	2	1	1	0.95
Reoperation (*n*)	2	2	3	0.305
Artery thrombosis (*n*)	1	0	0	0.582
Portal vein complication (*n*)	2	2	0	0.531
Biliary complication (*n*)	1	2	0	0.38
Gastrointestinal complications (*n*)	1	1	1	0.822
Infection (*n*)	10	1	4	0.102
Ascites (*n*)	4	0	0	0.112
Anemia (*n*)	2	0	0	0.337
MODS (*n*)	4	0	0	0.112

*Significant difference between ODH and PLDH; ^#^significant difference between PLDH and FAPLDH; ^&^significant difference between ODH and FAPLDH. Values are mean (±S.D.) unless indicated otherwise. BMI, body mass index; GRWR, graft recipient weight ratio; ALB, albumin; ALT, alanine aminotransferase; AST, aspartate aminotransferase; TB, total bilirubin; PT, prothrombin time; INR, international normalized ratio; ICU, intensive care unit; MODS, Multiple Organ Dysfunction Syndrome.

### Basic Characteristics and Pre-operative Data of Donors

More female donations were performed in the PLDH group (male: female 20:70 *vs*. 61:76; *p*=0.001) and FAPLDH group (12:46 *vs*. 61:76; *p* = 0.002) than those in the ODH group. Mean weight (57.12 ± 10.21 kg *vs*. 61.54 ± 11.71 kg; *p* = 0.010) and BMI (21.39 ± 3.13 *vs*. 22.33 ± 2.81; *p* = 0.049) were lower in the PLDH group than in the ODH group. There were no statistical differences in age, height and laboratory data among the PLDH, FAPLDH and ODH donor groups (Table 1).

### Intraoperative Donor Characteristics

There were no intraoperative deaths in either group. The operation time was 169.29 ± 26.68 min in the PLDH group, which was longer than 154.34 ± 18.40 min in the FAPLDH group (*p* = 0.001) and 146.08 ± 25.39 min in the ODH group (*p* = 0.001). The FAPLDH was associated with significantly lower blood loss (39.48 ± 10.46 mL), compared with that in the PLDH group (52.44 ± 18.44 mL; *p* = 0.019) and that in the ODH group (108.80 ± 36.82 mL, *p* = 0.001). Also, blood loss in the PLDH group was less than in the ODH group (*p* = 0.001). The graft was 270.25 ± 71.33 g in the ODH group, which was heavier than 239.46 ± 41.07 g in the PLDH group and 241.38 ± 42.22 g in the FAPLDH group (*p* = 0.001). None of the donors received blood transfusion.

### Post-operative Outcome of Donors

There were no significant differences among groups on post-operative peak liver function indexes (ALT, AST, TB, PT and INR). However, the PLDH group had a significantly longer post-operative hospitalization (*p* = 0.001). There was no death in any group and only one bile leakage complication (≥Clavien- Dindo II), which was managed without surgical intervention in the ODH group. Complications ≤ Clavien-Dindo I were not included in our statistics, such as post-operative low fever and incision pain.

### Outcome of Recipients

The basic characteristics, liver function information and post-operative complications of the pediatric recipients are summarized in Table 2. There were no significant differences between their age, height, weight, BMI, sex, GRWR, liver function and post-operative complications. Four cases of perioperative death were observed, due to severe post-operative MODS (ODH) and infection (PLDH and FAPLDH). Seven reoperations were done due to gastrointestinal complications (perforation and intestinal obstruction) and infections. For the remaining post-operative complications, conservative symptomatic treatment, or interventional treatment (such as stenting for PVT) were adopted.

### Quality of Life in Donors

For SF-36 questionnaire, a total of 337 donors completed the questionnaire validly, including 218 donors for open surgery and 119 donors for laparoscopic surgery (Table 3). The results of this questionnaire reflect the influence of different surgical methods on the long-term quality of life of donors. Overall, we found that laparoscopic donors had higher long-term quality of life than open donors. There were significant differences in the scores of the five items related to physical functioning and one item of mental health, with higher scores in the laparoscopic group. In the final total score, physical functioning in the laparoscopic group was significantly better than that in the open group.

**Table 3:** The results of SF-36 questionnaire of donors.

**Analyzed factors**	**ODH (*n* = 218)**	**PLDH (*n* = 119)**	***p*-value**
Sex (male:female)	65:153	29:90	0.287
Age (years)	33.65 ± 6.158	32.6 ± 6.023	0.131
HT	3.14 ± 0.927	3.20 ± 1.094	0.616
PF1	2.38 ± 0.665	2.43 ± 0.619	0.475
PF2	2.87 ± 0.374	2.92 ± 0.334	0.265
PF3	2.89 ± 0.375	2.93 ± 0.312	0.318
PF4	2.80 ± 0.414	2.83 ± 0.397	0.468
PF5	2.96 ± 0.232	2.98 ± 0.129	0.313
PF6	2.88 ± 0.382	2.97 ± 0.181	0.004*
PF7	2.75 ± 0.520	2.86 ± 0.375	0.034*
PF8	2.86 ± 0.422	2.95 ± 0.220	0.009*
PF9	2.96 ± 0.251	3.00 ± 0	0.032*
PF10	2.96 ± 0.251	3.00 ± 0	0.032*
RP1	1.86 ± 0.345	1.89 ± 0.313	0.457
RP2	1.87 ± 0.340	1.92 ± 0.279	0.156
RP3	1.84 ± 0.368	1.89 ± 0.313	0.178
RP4	1.78 ± 0.412	1.78 ± 0.415	0.951
RE1	1.81 ± 0.392	1.83 ± 0.376	0.65
RE2	1.83 ± 0.372	1.83 ± 0.376	0.945
RE3	1.77 ± 0.424	1.84 ± 0.368	0.095
SF1	4.48 ± 0.810	4.48 ± 0.636	0.982
SF2	5.43 ± 1.110	5.46 ± 1.023	0.801
BP1	5.62 ± 0.562	5.70 ± 0.427	0.146
BP2	5.16 ± 1.013	5.28 ± 0.883	0.305
VT1	4.00 ± 1.678	4.14 ± 1.653	0.468
VT2	3.91 ± 1.455	3.95 ± 1.615	0.837
VT3	4.65 ± 1.229	4.79 ± 1.127	0.294
VT4	4.81 ± 1.130	4.87 ± 1.157	0.68
MH1	4.17 ± 1.517	4.59 ± 1.417	0.015*
MH2	4.94 ± 1.254	5.06 ± 1.174	0.379
MH3	3.83 ± 1.580	3.92 ± 1.675	0.66
MH4	4.78 ± 1.136	5.02 ± 0.991	0.052
MH5	4.08 ± 1.479	4.16 ± 1.573	0.655
GH1	3.54 ± 1.074	3.68 ± 0.892	0.189
GH2	3.82 ± 1.208	3.74 ± 1.131	0.56
GH3	4.01 ± 1.086	4.11 ± 0.946	0.399
GH4	3.95 ± 1.131	3.87 ± 1.117	0.533
GH5	4.00 ± 1.032	4.00 ± 0.902	0.968
PF SUM	91.58 ± 14.057	94.33 ± 7.917	0.022*
RP SUM	83.83 ± 31.559	86.97 ± 25.803	0.325
BP SUM	87.78 ± 15.171	89.78 ± 12.566	0.222
GH SUM	71.56 ± 22.072	72.02 ± 19.640	0.845
VT SUM	66.88 ± 18.969	68.74 ± 19.136	0.392
SF SUM	79.08 ± 14.999	79.41 ± 13.422	0.842
RE SUM	80.43 ± 34.231	83.47 ± 30.026	0.398
MH SUM	67.21 ± 18.119	70.96 ± 18.714	0.074
HT SUM	53.56 ± 23.182	55.04 ± 27.344	0.616

Values are mean (±s.d.). *Statistically significant. SUM refers to the final score calculated from the corresponding item. HT, Reported Health Transition; PF, Physical Functioning; RP, Role-Physical; RE, Role-Emotional; SF, Social Functioning; BP, Bodily Pain; VT, Vitality; MH, Mental Health; GH, General Health.

## Discussion

In 2002, Cherqui et al. firstly reported (15) the feasibility of laparoscopic left lateral resection for liver transplantation in children. Because of its safety and reproducibility, the laparoscopic approach was recommended as a new standard practice for obtaining left lobe for liver transplantation (24). After the Louisville Statement in 2008 (25), the application of laparoscopy in liver surgery has grown rapidly. However, the efficacy of laparoscopic donor hepatectomy for liver transplantation remains controversial, due to no safety differences between laparoscopic and open surgery in highly specialized centers, and the lack of evidence on long-term outcomes for donors and recipients (12).

Due to the unique staining characteristics of ICG, our team innovatively combined ICG fluorescence technique with laparoscopic donor liver harvest. Functional staining of the liver by ICG enhances the exposure of bile ducts and blood vessels in the liver to the surgeon. Combined with the magnification effect, laparoscopy with ICG fluorescence assistance can theoretically significantly reduce intraoperative blood loss and lower the risk of post-operative biliary complications, which were confirmed by our data analysis.

PLDH requires a team with professional experience of both liver transplantation and laparoscopic surgery, however only a few medical centers can perform large-scale of PLDH procedures. At Shanghai Renji Hospital, more than 300 LDLT are performed every year, of which open, laparoscopic and fluorescence-assisted surgeries are included. All donor operations in this study were performed by the same surgical team to avoid technique-induced bias.

When analyzing the data from this retrospective study, we found that the proportion of female in the PLDH group and the FAPLDH group was significant larger. We believe that this is related to the cosmesis effect and less post-operative incision pain brought by laparoscopic surgery (19), so minimally invasive laparoscopic LDLT is more favored by female. In addition, we believe that the differences in body weight and BMI are to a large extent caused by gender differences in each group, because females generally wish to have a lower body weight and BMI in China.

Regarding the amount of intraoperative blood loss, the FAPLDH group was significantly less than the other two groups, which was consistent with our previous prediction. Fluorescence staining indeed effectively helped the operator observe the anatomical position of liver blood vessels, so as to avoid the damage of blood vessels leading to massive bleeding. The amount of blood loss in the PLDH group was also significantly lower than that in the ODH group, which seems to have been the unified conclusion of related research groups (16–19), even though some studies showed no difference in the amount of blood loss (26–30). This is due to the magnification of the laparoscope, which allows the surgeon to perform more accurately, and the pressure of the pneumoperitoneum, which reduces the outflow of blood. Therefore, we believe that the reduction of blood loss improves the safety of LDLT.

Secondly, the operation time of PLDH group was significantly longer than that of ODH group. We believe that the main reason for this result is the preparation of laparoscopy and pneumoperitoneum and the duration of mobilization. However, the operation time of the FAPLDH group was close to that of the ODH group, which was unexpected. In theory, ICG preparation should prolong the operation. Therefore, we hypothesized that ICG fluorescence accelerated the anatomical location, intraoperative dissection of blood vessels and bile ducts (31). Laparoscopic hepatectomy assisted by ICG speeds up the completion of the operation, reduces the risk of anesthesia and improves the safety of the operation. But we did not calculate the damage to the donor caused by ICG itself. Adverse reactions are reported with a frequency of 0.003% under the dose of about 0.5 mg/kg bodyweight (32), while over the maximum dose of 2 mg/kg bodyweight a low frequency of adverse events including anaphylaxis or mortality could be observed (33). Fortunately, very few adverse reactions or toxicity have been reported with our ICG dose. Therefore, we believe that hepatectomy intraoperative ICG dose does not cause health effects in healthy donors. Additionally, we applied two-dimensional camera system in our donor surgery with no experience of three-dimensional one which is reported to contributes to accurate hilum dissection, better identification of vessels and ducts, and convenience of hemostasis. Three-dimensional visualization in combination with fluorescent image assistance will have additional benefits in donor surgery by providing better depth perception and tactile feedback.

Interestingly, the graft weight of the ODH group was greater than that of the other two groups, we thought the open procedure was more conducive to obtaining a larger liver. Then we added GRWR, and it was found that GRWR of ODH group was the smallest although have no significance. We thought the difference in graft weight was due to the difference in the weight of recipient. Although not significant, the ODH group did have higher receptor weight than the other two groups.

The difference in length of hospital stay was statistically significant, but we did not think it was clinically significant. For LDLT donors, we usually take a hospital stay of 4–5 days, which can be extended appropriately if necessary, according to the donor's wishes, rather than the need to be discharged immediately after the donor recovers. Post-operative liver function and blood coagulation suggest that there is no difference in the short-term prognosis of the donor between the three operations. In terms of complications, we did not include complications in the statistics because the complications (≥Clavien- Dindo II) of liver transplantation donors in our hospital were relatively rare, most of which were post-operative low fever and incision pain, which could be recovered during hospitalization.

We then followed up the open and laparoscopic donors with SF-36 questionnaires at least 1 year post-operatively (34). The final scores of the SF-36 questionnaire were significantly different between the two groups. Donors in the laparoscopic group had significantly higher Physical Functioning scores than those in the open surgery group, indicating that the laparoscopic surgical method had less impact on the daily activities of the donors, which may be related to smaller incisions and less incision pain. The final Mental Health scores in the laparoscopic group were also higher than those in the open group, although not to a significant extent (*p* = 0.07). We think this difference may be due to less psychological stress on the donor with the more aesthetically pleasing smaller incision, and they are more psychologically receptive to the outcome. This suggests that pure laparoscopic donor hepatectomy has at least the same long-term prognosis and quality of life as traditional open surgery, and may even be superior. In addition, short-term outcomes of the recipients were also briefly analyzed and no significant differences were found.

At last, the recipients are Pediatric age group and generally would need a smaller liver segment (left lateral and probably more feasible) compared to adult recipients patients and hence application of such techniques in acquisition of larger liver segments from the donor for adult-to-adult LDLT is achievable (35, 36).

## Conclusion

In summary, we conclude that pure laparoscopic donor hepatectomy with/without fluorescent-assist has at least the same outcome and prognosis as traditional open surgery. In some respects, especially the amount of blood loss, laparoscopic surgery has significant advantages. The overall quality of life of laparoscopic donors was higher than that of open donors.

## Data Availability Statement

The raw data supporting the conclusions of this article will be made available by the authors, without undue reservation.

## Ethics Statement

The studies involving human participants were reviewed and approved by the Institutional Review Board of Renji Hospital affiliated to Shanghai Jiao Tong University School of Medicine (IRB Reference Number: KY2020-190). Written informed consent to participate in this study was provided by the participants' legal guardian/next of kin.

## Author Contributions

KH, YL, and QX: conception, design, and surgeries. QX: administrative support. YP and HW: collection and assembly of data. KH, YP, and HW: data analysis and interpretation. All authors provision of study materials or patients and manuscript writing.

## Funding

This study was supported by the Project of the Shanghai Municipal Health Commission (20204Y0012), Innovative Research Team of High-Level Local Universities in Shanghai (SSMU-ZDCX20180802), National Natural Science Foundation of China (81972205), the Project of Shanghai Key Clinical Specialties (shslczdzk05801), Seed Fund of Renji Hospital (RJZZ18-010), and Shenkang 3-year action plan (SHDC2020CR2003A, SHDC2020CR5012).

## Conflict of Interest

The authors declare that the research was conducted in the absence of any commercial or financial relationships that could be construed as a potential conflict of interest.

## Publisher's Note

All claims expressed in this article are solely those of the authors and do not necessarily represent those of their affiliated organizations, or those of the publisher, the editors and the reviewers. Any product that may be evaluated in this article, or claim that may be made by its manufacturer, is not guaranteed or endorsed by the publisher.

## Abbreviations

LDLT: living donor liver transplantation
PLDH: pure laparoscopy donor hepatectomy
FAPLDH: fluorescence-assisted pure laparoscopy donor hepatectomy
ODH: open donor hepatectomy
ICG: indocyanine green
GRWR: graft recipient weight ratio
BMI: body mass index
ALT: alanine aminotransferase
AST: aspartate aminotransferase
TB: total bilirubin
PT: prothrombin time
INR: international normalized ratio
PVT: portal vein thrombosis
MODS: multiple organ dysfunction syndrome.
